# Purification and Partial Characterization of β-Glucosidase in Chayote (*Sechium edule*)

**DOI:** 10.3390/molecules201019372

**Published:** 2015-10-23

**Authors:** Sergio Espíndola Mateos, Carlos Alberto Matías Cervantes, Edgar Zenteno, Marie-Christine Slomianny, Juan Alpuche, Pedro Hernández-Cruz, Ruth Martínez-Cruz, Maria del Socorro Pina Canseco, Eduardo Pérez-Campos, Manuel Sánchez Rubio, Laura Pérez-Campos Mayoral, Margarito Martínez-Cruz

**Affiliations:** 1 Unidad de Bioquímica e Inmunología, Instituto Tecnológico de Oaxaca, Oaxaca 68030, Mexico; E-Mails: sespindolam@hotmail.com (S.E.M.); carloscervantes.ox@outlook.com (C.A.M.C.); perezcampos123@yahoo.es (E.P.-C.); jmsanchezrubio@gmail.com (M.S.R.); 2 Centro de Investigación Medicina-UNAM-UABJO, Facultad de Medicina y Cirugía, Universidad Autónoma “Benito Juárez” de Oaxaca, Oaxaca 68050, Mexico; E-Mails: juan_alpuche@hotmail.com (J.A.); fuegoblanco68@yahoo.com.mx (P.H.-C.); rmc.azul@gmail.com (R.M.-C.); socopina12@hotmail.com (M.S.P.C.); laurapcm@prodigy.net.mx (L.P.-C.M.); 3 Facultad de Medicina de la, Universidad Nacional Autónoma de México, Distrito Federal 04510, Mexico; E-Mail: ezenteno@servidor.unam.mx; 4 Unité Mixte de Recherche CNRS/USTL 8576, Glycobiologie Structurale et Fonctionnelle, Université des Sciences et Technologies de Lille 1, Villeneuve d’Ascq 59655, France; E-Mail: Marie-Christine.Slomianny@univ-lille1.fr

**Keywords:** β-glucosidase, *Cucurbitaceae*, *Sechium edule*, glycosyl hydrolases

## Abstract

β-Glucosidase (EC 3.2.1.21) is a prominent member of the GH1 family of glycoside hydrolases. The properties of this β-glucosidase appear to include resistance to temperature, urea, and iodoacetamide, and it is activated by 2-ME, similar to other members. β-Glucosidase from chayote (*Sechium edule*) was purified by ionic-interchange chromatography and molecular exclusion chromatography. Peptides detected by LC-ESI-MS/MS were compared with other β-glucosidases using the BLAST program. This enzyme is a 116 kDa protein composed of two sub-units of 58 kDa and shows homology with *Cucumis sativus* β-glucosidase (NCBI reference sequence XP_004154617.1), in which seven peptides were found with relative masses ranging from 874.3643 to 1587.8297. The stability of β-glucosidase depends on an initial concentration of 0.2 mg/mL of protein at pH 5.0 which decreases by 33% in a period of 30 h, and then stabilizes and is active for the next 5 days (pH 4.0 gives similar results). One hundred μg/mL β-d-glucose inhibited β-glucosidase activity by more than 50%. The enzyme had a *K*_m_ of 4.88 mM with *p*-NPG and a *K*_cat_ of 10,000 min^−1^. The optimal conditions for the enzyme require a pH of 4.0 and a temperature of 50 °C.

## 1. Introduction

β-Glucosidase (EC 3.2.1.21) is responsible for the hydrolysis of terminal non-reducing glycosyl residues in oligosaccharides and glycosides [1]. It occurs widely in prokaryotes and eukaryotes, and is involved in various functions in plants, such as lignification [2,3] and cell wall β-glucan turnover [4,5,6]. In chemical defense, it blocks the action of pathogens and herbivores [7,8], regulates the biological activity of cytokinins, activates phytohormones [9], and releases the scent in plants [10,11,12]. Hydrolysis of glycosidic linkages in secondary metabolites, such as, cyanogenic flavonoid and hydroxamic acid glycosides, can drastically alter biological activity, chemical stability, and the solubility of the molecule [13,14,15].

Plants have the largest number of glycosyl hydrolase (GH1) family proteins, for example, 40 GH1 genes are found in rice genome sequences, two of which appear to be endophyte genes. Similarly, the presence of 26 isoforms have been reported in maize [16].

Varieties of *Cucurbitaceae* genus contain β-glucosidases: *Cucumis sativus* (cucumber) has a predictive sequence for β-glucosidase 24 (511 amino acid, NCBI Reference Sequence: XP_004154617.1); *Cucumis* *melo* (melon) (150 aa. NCBI Reference Sequence: AFO12658.1); *Citrullus lanatus* (watermelon) (327 aa, NCBI Reference Sequence: AEL33712.1); and *Cucurbita pepo* (zucchinni pepo), among others. The natural substrate specificity for the molecules in these plants is not known, however, Poungbangpho *et al.* reported β-d-galactosidase, β-d-glucosidase, and α-d-mannosidase in seeds of *Cucurbiataceae*, using pNP-β-d-galactopyranoside, pNP-β-d-glucopyranoside and pNP-α-mannopyranoside as substrates [17].

In general, enzymes are used as catalysts for energy production, and for pharmaceutical or industrial food purposes, due to their efficiency under mild conditions, such as low temperatures, changes in pressure, and in aqueous solutions. However, in contrast to these benefits, the enzymes have certain disadvantages, such as low or poor stability, making them unpredictable in industrial contexts.

One disadvantage associated with the stability of multimeric enzymes, such as dehydrogenases, aldolases, oxidases, catalases and hydrolases (e.g., β-glucosidase), is dissociation of the subunits, and consequently, with the molecular organization, resulting in reduced activity. However, this characteristic is not associated with low stability, other factors can affect the dissociation, such as temperature, pH, concentration, cofactors, and metal ions. Furthermore, under certain conditions, the interactions between sub-units weaken and dissociate, specifically in industrial applications [18,19,20].

It should be noted that β-glucose is the product in the hydrolysis of β-glucosidases. β-glucose, mannose, xylose, and galactose do not prevent the inhibitory effect over enzymes, such as β-glucosidaes and glucanase. This indicates that the industrial applications of β-glucosidase may be stabilized by the immobilization of these enzymes. Immobilization is known to exclude other polymers and sugars and improve the effects of the protein, resulting in thermodynamic stability [21,22].

The present study evaluated a new β-glucosidase from *Cucurbitaceae* genus that was purified and partially characterized. The properties of purified β-glucosidase in *Sechium edule* are discussed below. 

## 2. Results and Discussion

### 2.1. Enzyme Purification

A yield of 0.67% protein (134 mg/200 g), with 2.92 U/mg specific activity was obtained from the crude extract, and 52.14 mg of protein was obtained from precipitation, which had a specific activity of 5.45 U/mg (Table 1).

**Table 1:** Purification chart for β-glucosidase in *Sechium edule*, from 200 g of pulp, homogenized in acetate buffer at pH 5.0.

Fraction	Total Protein (mg)	Activity of β-Glucosidase (U)	Specific Activity (U/mg)	Purification	Yield (%)
Crude extract	134.37	393.45	2.92	1	100
Protein precipitated	52.14	285.62	5.47	1.87	72.59
CMCEL Fraction I	12.50	198.81	15.9	5.44	50.5
CMCEL fraction II	3.16	128	40.5	13.86	32.53
Sephacryl- Fraction IV	2.46	155.27	63.11	21.61	39.46

At the first stage of purification, elutions of protein from two fractions (as recorded at 280 nm) presented enzymatic activity through cationic interchange chromatography.

**Figure 1:**
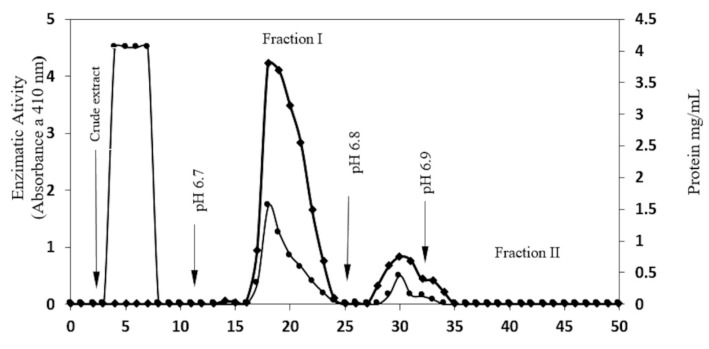
Purification profile of β-glucosidase in *S. edule*. CMC column, after equilibration with acetate buffer (0.2 M, pH 5), 50 mg of protein from crude extract was applied. The column was washed with acetate buffer until O.D at 280 nm was <0.01, and then eluted using a phosphate buffer at ph 6.7, 6.8, and 6.9. Flow was 35 mL/h, 1 mL fraction was collected. Enzymatic activity (♦─♦) and protein concentration (●─●) was determined for each fraction.

In Table 1, Figure 1, Fraction I (pH 6.7) shows 12.5 mg of protein as 199 U/β-glucosidase activity, and 15.9 U/mg as specific activity.

**Figure 2:**
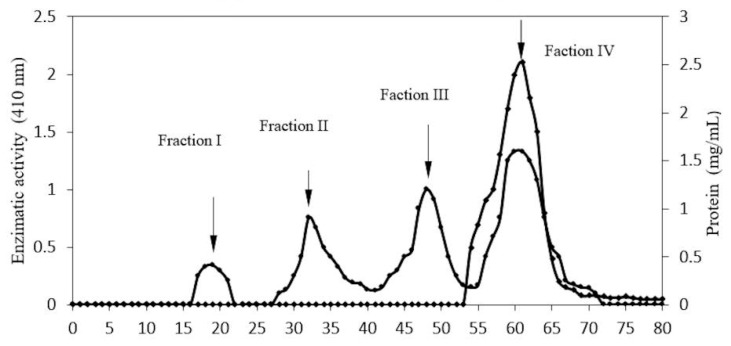
Profile of purification of β-glucosidase in *S. edule*. Gel filtration Sephacryl S-200HR, equilibrated using phosphate buffer (0.2 M, pH 6.7). 5 mg of protein was applied and eluted at flow rate of 16 mL/h, collecting 1.5 mL fractions. Protein concentration (●─●) and enzymatic activity (♦─♦) was taken for each fraction.

Fraction II (pH 6.8) shows 3.16 mg of protein with 128 units of β-glucosidase and 40.5 U/mg as specific activity. Fraction I was chosen at the next stage of purification, having the highest level of glucosidase activity. Of the four protein fractions obtained, fraction IV presented greater enzymatic activity (Figure 2). This fraction showed 2.46 mg of protein, 155 units as glucosidase activity, and 63 U/mg as specific activity, as shown in Table 1. This method shows the purification of a fraction of protein with 21.61 times more specific activity than the crude extract (Table 1).

As shown in Figure 2, the enzyme was purified in an additional stage through gel filtration. The sample obtained from the ion exchange column contained contaminants with higher and lower molecular weights than for β-glucosidase. 

At this stage of the purification process some separated contaminants may act as natural substrates, *i.e.*, glucosides or polysaccharides, which contain glucose. Their release of contaminants in the filtration process permits an increase in enzymatic activity.

**Figure 3:**
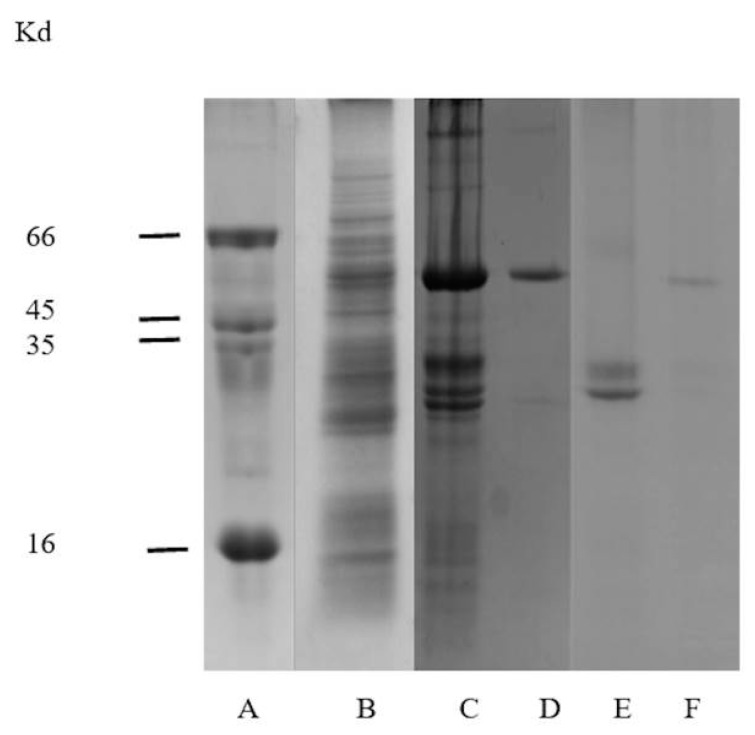
SDS-PAGEs from the purification process of *S. edule*. Molecular weight (Sigma-Aldrich, St. Louis, MO, USA) was used on Lane **A**: bovine serum albumin (66 kDa), ovalbumin (45 kDa), pepsin (35 kDa), and tripsin inhibitor (16 kDa); Lane **B**: Protein precipitation of crude extract; Lane **C**: Eluted fraction I (pH 6.7) from CMC column; Lane **D**: Eluted fraction two (pH 6.8) from CMC column; Lane **E**: Fractions I-III from size exclusion chromatography; Lane **F**: Fraction IV Sephacryl S-200HR chromatography (Sigma-Aldrich) purified protein with glucosidase activity.

A basic procedure for the isolation and purification of a β-glucosidase from chayote (*Sechium edule*) is described in this study. The most effective environment for protein precipitation required a pH 4.6, which resulted in 72% soluble protein, re-solubilized at pH 5.0. Assays were achieved using fruit pulp, which gave a very low protein solubility (Table 1). The conditions for purification were similar to those of β-glucosidase in maize [23], to which a low isoelectric point has been attributed. The isoelectric point of purified protein in *S. edule* was between 6.7 and 6.8, according to data from the elution profile in the ionic interchange column (Figure 1, Figure 3 lines C and D).

The majority of β-glucosidase was precipitated from the crude extract; however, just 2% of enzymatic activity was found in the supernatants. Similarly, no protein precipitation was found between pH 7.0 and 8.5, and the highest enzymatic activity was found with acidic conditions. The elution profile in the ion interchange column gives two components at pH 6.7 and 6.8. Both fractions presented glucosidase activity, suggesting the presence of at least two isoforms, with similar electrophoretic mobility from the enzyme, such as those reported in enzymes of grass plants, *i.e.*, maize, barley, sorghum and rice [23,24,25,26,27,28,29]. With regard to maize, Tiessen *et al.* presented a wide range of information related to 26 possible isoforms of β-glucosidase which suggests that more than one gene is involved in protein codification [16]. The presence of β-glucosidase isoforms is due to the presence of glycosylation, but, more importantly, could also be attributed to the nature of amino acids and differences in their electrical charges.

### 2.2. Electrophoretic Analysis

**Figure 4:**
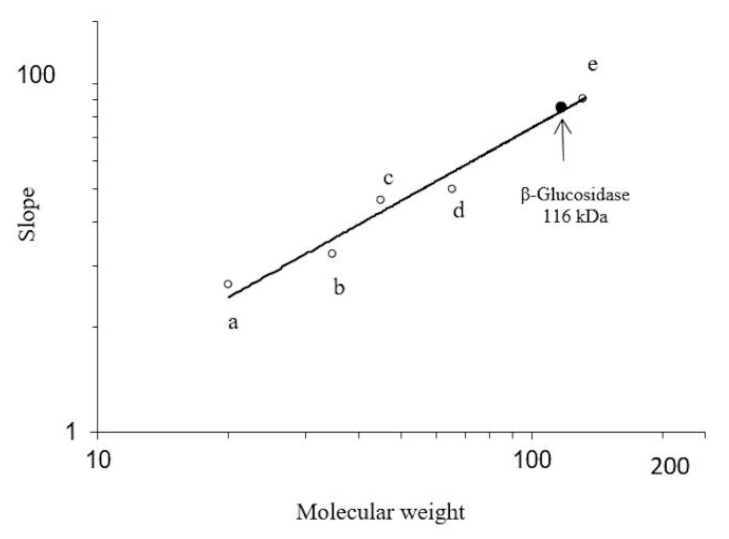
Determination of molecular weight of β-glucosidase in *Sechium edule* from non-denaturing conditions. Electrophoresis on gels at 5%, 7.5%, 10% and 12.5% polyacrylamide concentrations, at pH 8.3 were performed, and the relative R*_f_* of the proteins in each gel, relative to the tracking dye (bromophehenol blue), was plotted. Molecular weight standards were: (a) α-lactalbumin (14,200); (b) carbonic anhydrase (29,000); (c) ovalbumin (45,000); (d and e) bovine serum albumin (monomer, 66,000; and dimer 132,000).

All fractions obtained were analyzed by SDS PAGE (Figure 3). Initially, the fractions showed a combination of proteins. However, after continued purification, 58 kDa protein showed glucosidase activity and a molecular mass of 116 kDa was obtained through native PAGE analysis. As shown in Figure 4.

Electrophoretic analysis of fractions eluted at pH 6.7 in Figure 3, line C, show several proteins above and below a richer band of 58 kDa, which is also presented as crude extract, (Figure 3, line B). This appears to correspond to the β-glucosidase band obtained from the fraction at pH 6.8 in Figure 3, line D which expresses high glucosidase activity 40.5 U/mg, Table 1. Additionally, component one, of Figure 3, line C, which corresponds to a fraction contaminated by several proteins, shows a richer band of possible β-glucosidase purified by size exclusion chromatography. At the end of the purification process, 98% of enzymatic activity was found in fraction IV, (Figure 2). This fraction represents a 116 kDa protein (as determined by native-PAGE. Figure 4), indicating that the β-glucosidase obtained is a homodimeric protein with an apparent molecular weight of 58 kDa subunits (Figure 3, line F). Analyses of peaks with lower molecular mass, containing contaminant proteins (I, II and III), presented no enzymatic activity (Figure 3, line E). Further analysis of the electrophoretic pattern shows a band with a similar molecular mass, 58 kDa during the purification process (Figure 1 and Figure 3). This confirms the presence of two isoforms, which can be eluted at various pH(s). The presence of isoforms is a frequent phenomenon in protein molecules as they participate in similar functions, the only difference being an affinity to various substrates [16,17,18,19,20,21,22,23,24,25,26,27,28]. Such phenomena are present in Glu1 and Glu2 in maize. For example, Glu2 does not hydrolyze artificial substrates commonly used in Zymograms, unless 4-methylumbelliferyl-β-d-glucosyl is used [30]. Furthermore, Glu2 is less efficient (5 to 6 times) than Glu1 in hydrolysis of nitrophenyl glycosides and it does not hydrolyze 6-bromo-2-naphtyl-β-d-glucoside which is readily hydrolyzed by Glu1 [30,31]. The data strongly suggests that the isoelectric point (pI) for β-glucosidase in *S. edule* ranges from 6.7 to 6.8, which is at odds with the pI of previous β-glucosidase results, e.g., cucumber pI 9.27, (accession gi|700211355). 

### 2.3. Peptide Analysis by Nano-LC-ESI-MS/MS

The purified enzyme (58 kDa) was excised from the SDS-PAGE and analyzed by ESI, as described previously [32,33]. Analysis of the triptych peptides showed similarities to β-glucosidase 24-like in *Cucumis sativus* (cucumber: XP_004154617.1), which possesses a calculated molecular mass of 58.142 kDa. Searches performed by the BLAST program found seven peptides with relative masses ranging from 874.3643 to 1587.8297 (protein score 62), covering 10% of the sequence for glycosyl hydrolase, GH I (Table 2).

The analysis of triptych peptides in the 58 kDa subunit from β-glucosidase in *S. edule* exhibited little similarity in the amino acid sequences, relative to other β-glucosidases existing in the data bases. In this study, low homology was found in the β-glucosidase in cucumber (*Cucumis sativus*) and the seven peptides in *S. edule*, which were related when both enzymes were compared. This protein, (XP_004154617.1) was reported to have a predictive sequence of 511 amino acids and a calculated molecular mass of 58.142 kDa. Two of the seven peptides––FSIAWSR and MGFDAYR––were identified in the amino terminal residues, 107 to 113 and 114 to 120, respectively. In the middle of the sequence of the DFSEVCFK peptide residue, 184 to 191, four sequences in the carboxyl terminal region were found: EAMQAGVRVK from residue 453 to 462, FGLTYIDYK from residue 483 to 491, NNLK from residue 492 to 495, and a terminal peptide WFENFLK from 504 to 510 (Table 2).

**Table 2:** Analysis of tryptic peptides from the 58 kDa subunit *of Sechium edule* β-glucosidase (Sebg) by nano-LC-ESI-MS/MS.

Name	Mr ^a^	Delta ^b^	Peptide ^c^	Start-End ^d^ Sequence
Sebg1	874.3643	0.0196	MGFDAYR	107-113- Csbg
Sebg2	865.446	0.0133	FSIAWSR	114-120- Csbg
Sebg3	982.4912	−0.0026	WFENFLK	504-510- Csbg
Sebg4	1030.4430	0.0092	DFSEVCFK	184-191- Csbg
Sebg5	1087.5808	−0.1337	EAMQAGVRVK	453-462- Csbg
Sebg6	1118.5648	−0.0072	FGLTYIDYK	483-491- Csbg
Sebg7	1587.8297	0.9914	NNLK	492-495- Csbg

^a^ Relative mass calculated. ^b^ Difference between experimental and calculated relative masses. ^c^ Peptide sequence homologous to *Cucumis sativus* (cucumber) β-glucosidase (Csbg). ^d^ Position of sequence in *Cucumis sativus* (cucumber) β-glucosidase.

The similarities in peptides in *Cucumis sativus* enzymes and *S. edule* amount to just 10%, thus highlighting an insufficient understanding of the complete sequence of this new enzyme, and concluding that these peptides represent a recognized protein with a molecular mass, similar to others already identified [15,16,17,18,19,20,21,22,23,24,25,26,27,28,29,30,31,32,33,34].

The amino acid sequence of the β-glucosidase reference was obtained from *Cucumis sativus*. β-glucosidase 24-like is a sequence obtained from the predictive database BLAST program, access number XP_004154167.1, with a 58,142 nominal molecular mass, similar to that identified in *S. edule*, which was calculated at 58 kDa. Table 2 compares the data for the homologous peptides found in both enzymes with a sequence of 10%. The low homology obtained from the BLAST database identifies just one member of the *Cucurbitaceae* family and is minimal. It is worth noting that β-glucosidase is a highly conserved enzyme in other families (as in the case of grasses), however, there is insufficient definitive information for this family. It is certainly a newly discovered β-glucosidase, which has not been reported previously. 

### 2.4. Optimal Conditions for β-Glucosidase Activity

**Figure 5:**
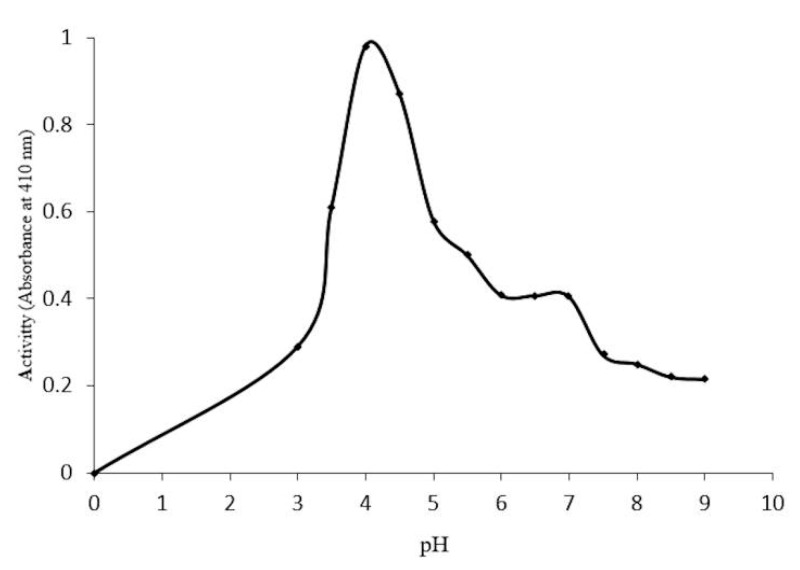
Effect of different pH(s) on the activity of the purified enzyme β-glucosidase in *Sechium edule.*

**Figure 6:**
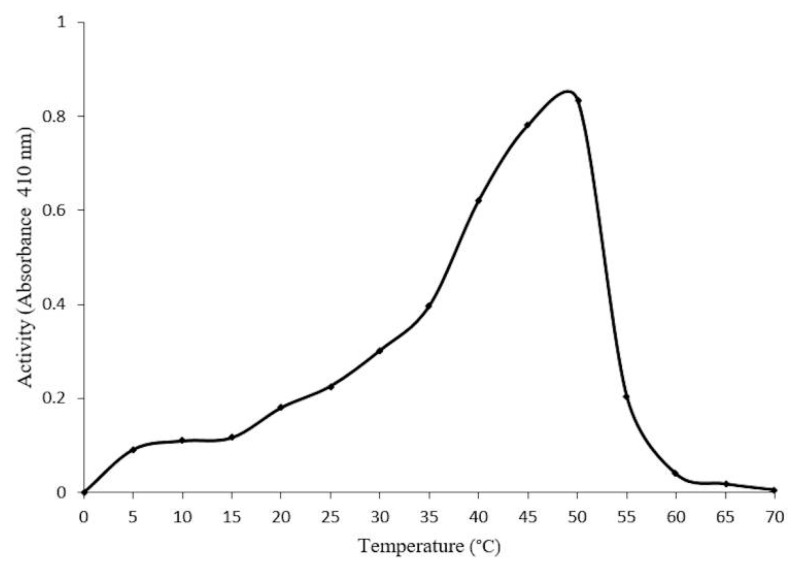
Effect of different temperatures on the activity of the purified β-glucosidase in *Sechium edule.* Maximum activity was observed at 50 °C, and a complete depletion of activity from 60 to 70 °C.

**Figure 7:**
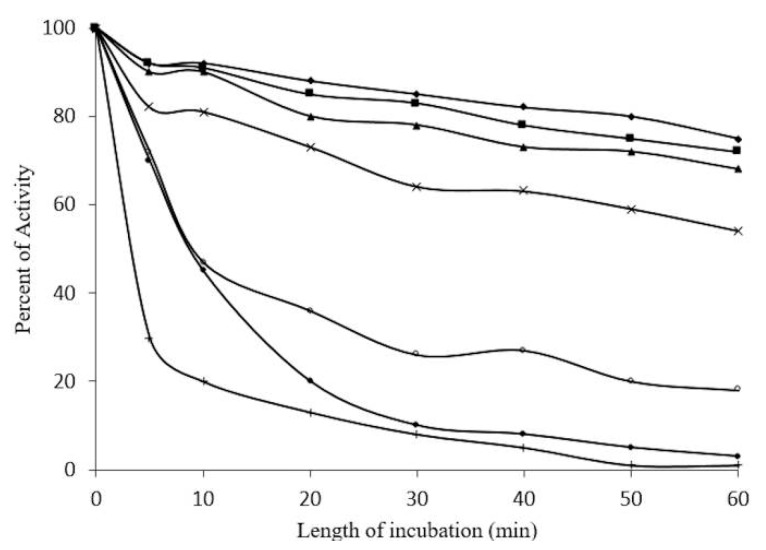
Thermostabilty of β-glucosidase (1 mg/mL) in Sechium edule *vs.* time of incubation (10 min intervals) at pH 5.0. Temperatures were ♦─♦ 30 °C, ■─■ 35 °C, ▲─▲ 40 °C, **x**─**x** 45 °C, ○─○ 50 °C,●─● 55 °C, +─+ 60 °C. Increasing temperatures and time of incubation lead to a complete loss of activity. (Please provide us original figures with high resolution).

Optimal conditions for enzyme activity require a pH of 4.0 (Figure 5), and a temperature of 50 °C (Figure 6). Thermostability assays indicate a 20%–30% reduction in enzymatic activity at 30–40 °C after 60 min. However, a 50% reduction in activity was found at 50–55 °C after 10 min, and a total loss of activity at 60 °C after 50 min (Figure 7).

**Figure 8:**
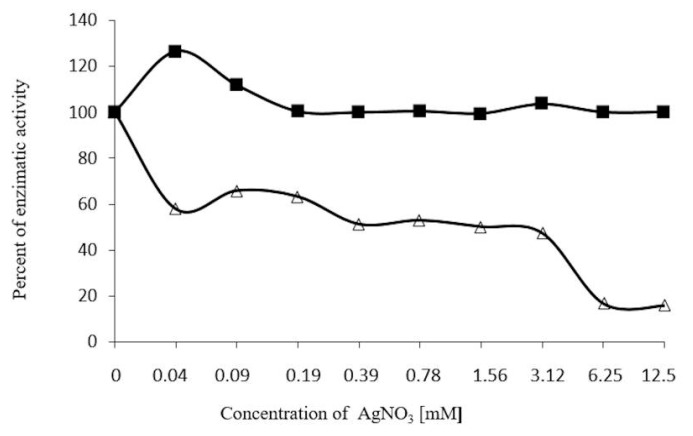
Effect of 2-mercaptoethanol on β-glucosidase, using different concentraions of AgNO_3_ (0.04 a 12.5 mM ∆─∆) as inhibitors (35 °C, 30 min), enzymatic activity was determined using *p*-nitrophenyl-β-glycopyranoside, and after incubating with 50 µL of 2-ME 100 mM ■─■ under the same conditions. Enzymatic activity was recorded at 410 nm.

**Figure 9:**
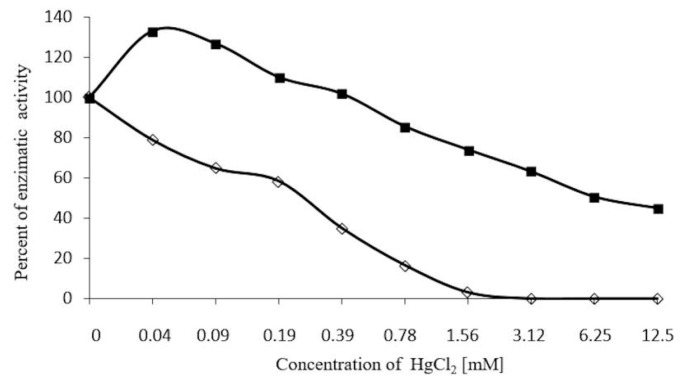
Effect of 2-mercaptoethanol on β-glucosidase , inhibited previously using HgCl_2_ at different concentrations (0.04 to 12.5 mM ◊─◊) at 35 °C, 30 min, enzymatic activity was determined using *p*-nitrophenyl-β-glycopyranoside, and after incubating with 50 µL of 2-ME 100 mM ■─■ under the same conditions. Enzymatic activity was recorded at 410 nm.

The enzyme inhibitor, Ag(+) was recorded at 6.25 mM (83% inhibition), and was reversed by incubating the enzyme with 100 mM of 2-ME (Figure 8). However, Hg2+ was more effective, as 0.78 mM inhibited 100% of enzyme activity. This activity was reversed by just 70%, using 2-ME 0.1 M (Figure 9). The ions, MgCl_2_ and ZnSO_4_ at 200 mM, failed to inhibit enzyme activity.

**Figure 10:**
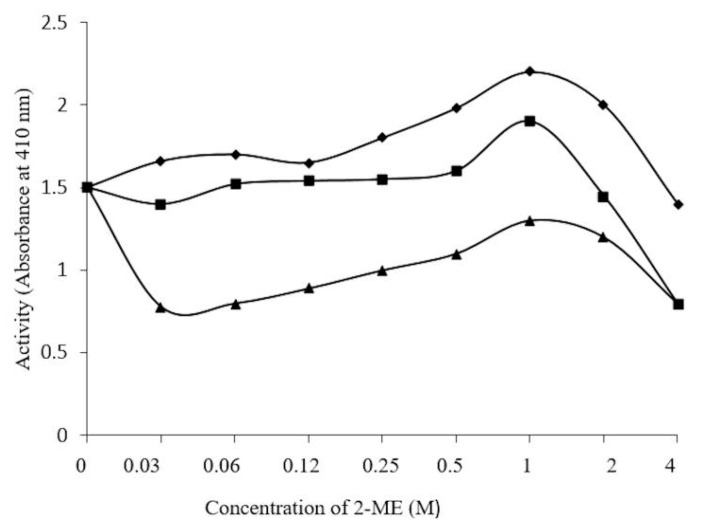
Profile of activity of β-glucosidase in the presence of different concentrations of a reducing agent (2-ME), incubated ♦─♦ 10 min, ■─■ 90 min, and ▲─▲ 24 h. Phosphate buffer, pH 5.0, and 1 mg/mL of purified enzyme, at 35 °C.

β-Glucosidase was incubated with various concentrations of 2-ME as a reduction agent, *i.e.* 0.03 to 4 M (Figure 10). Enzyme activity increased from 5% to 47% after 10 min. After 90 min, it showed an increment of 27%, and after 24 h, it was recorded at 50% to 86% of initial activity. 1 M of 2-ME improved the catalytic activity of this enzyme (Figure 10).

**Figure 11:**
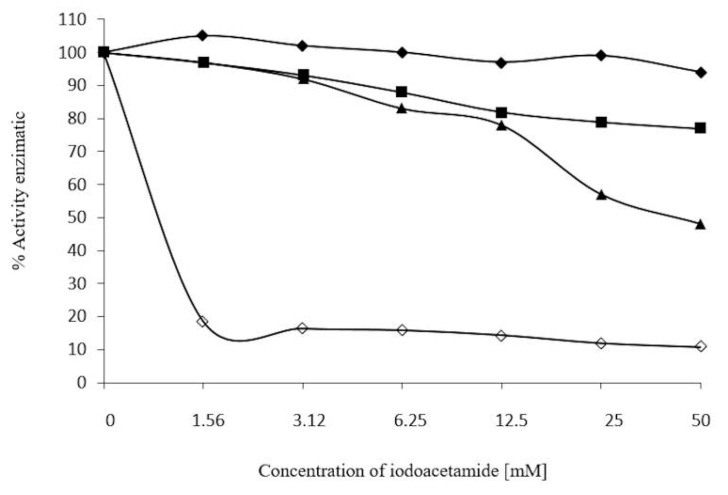
Effect of iodoacetamide on enzymatic activity of β-glucosidase in *S. edule.* Different concentrations and incubation times ♦─♦ 30 min, ■─■ 24 h, ▲─▲72 h, and ◊─◊ β-glucosidase. 1 M incubated previously in urea for 30 min. Enzymatic activity was determined using *p*-nitrophenyl-β-glycopyranoside, and recorded at 410 nm.

The SH groups of β-glucosidase were measured by using the alkylanting agent iodoacetamide, at 50 mM to 1.56 mM after 30 min, 24 h and 72 h. Little or no effect was found after 30 min, a 20% reduction in activity was found with 50 mM after 24 h, and an inhibition of 50% was reached after 72 h at 50 mM. Using urea as a denaturizing agent for proteins (1 M, 30 min) before iodoacetamide, the inhibiting effect was stronger, as was observed at 1.56 mM, with a reduction of 80% activity (Figure 11).

On the other hand, optimal pH activity registered 4.0, at 50 °C (Figure 5); these conditions were fairly similar to those for β-glucosyl hydrolases [15]. It is unlikely that this enzyme functions at optimal temperatures *in vivo* since this can lead to the inactivation of several other proteins, in which optimal activity is reported to be close to the denaturalization temperature. This is in accordance with the data, which shows 100% loss of enzymatic activity at 60 °C. In Figure 7, a loss of 90% activity was shown after 30 min, at 55 and 60 °C, and a complete loss of activity was recorded after 50 min, at 60 °C. The β-glucosidase in *S. edule* appears to have increased resistance for short periods at high temperatures, compared to maize and other β-glucosidades in grass and other plants, which have a loss of enzyme activity after 5 to 10 min [15,23]. This data strongly suggests that the enzyme is stable over long periods of time at the optimal temperature, due to a 70% reduction of activity in thermostability after 5 min, and is inactive for 50 min at 60 °C. This is in addition to the negligible resistance in maize as opposed to β-glucosidase, as previously reported by Esen *et al.* [22]. The enzyme activity was stable in the presence of 1 M 2-ME from 10 to 90 min, and was not inactivated at 4 M.

The loss of activity and instability of the protein structure induced by 2-ME indicated the presence of disulfide bonds in the enzyme, and suggests that these play a central role in native structures and on enzymatic activity. The high concentrations of 2-ME 4 M over 24 h reduce enzyme activity by 46% (Figure 10).

This may signify that the interaction of disulfide bonds with 2-ME is time dependent, and that a population of enzyme molecules affects oxidized sulfhydryl groups, and thus, is not catalytically functional. 2-ME activates these enzyme molecules by reducing their oxidized sulfhydryl groups, which are likely to be located on the surface of the enzyme. They are also more prone to reduction by 2-ME, exhibiting complete activity after incubation in the presence of 1 M 2-ME, after 10 to 90 min. It is clear that incubation for periods of time up to 24 h at relatively low concentrations (0.03 M) induces a transitory effect on inactivity, indicating the reduction of sulfhydryl groups of cysteines. This indicates a rearrangement of the tertiary structure of the enzyme, by adding increasing concentrations of 0.06 to 1 M 2-ME. There is an appropriate readjustment of disulfide bridges in the structure of the enzyme in order to reach maximum activity under these conditions. This is due to the progressive availability of the SH group participating in a more appropriate reorganization of the native enzyme structure [35,36]. The behavior of the β-glucosidases in *S. edule*, in the presence of 2-ME, indicates that the molecular structure is different to other β-glucosidases, since it needs longer incubation, responding with a decrease in initial activity. This is consistent with the low degree of homology found in other β-glucosidases, including cucumber (*Cucumis sativus*). It is likely that the natural substrate in this new β-glucosidase (chayotase) is different to others previously reported and needs to be studied further.

β-Glucosidase activity in plants is inhibited by ions, such as, Ag(+), Hg2+ (Ketuda Cairns [15]). In the case of β-glucosidase in *S. edule*, 83% inhibition is induced by AgNO_3_^+^ 6.25 mM, and is totally reversed when incubated with 0.1 M 2-ME, (Figure 8), while HgCl_2_ 1.56 mM completely inhibits the enzyme (Figure 9), recovering 70% of initial activity. When comparing the strong sensitivity of other β-glucosidases to ions, such as Ag(+) and Hg2+, as in *Zea mays*, it is worth noting that β-glucosidase in *S. edule* requires higher concentrations for inhibition, whereas, inhibition from Hg2+ is not completely reversed with 100 mM 2-ME.

These results suggest the interaction of Ag(+) with cysteines present in the vicinity or the binding site of the β-glucosidase. The Ag(+) ions react with the SH groups and the side group of cysteine residues in the protein chain, when there is an insufficient difference in electronegativity for a full ionic bond between silver and sulfur, in which case, the bond can be considered covalent. 

The interaction between Ag(+) and glucosidase displays hydrogen in disulfide, this interaction modifies the folding in the protein structure and consequently, inhibits the enzyme. Moreover, the complete reverse of activity in the presence of 2-ME 100 mM is attributed to a shift in the Ag(+) [37,38], which reconstructs the enzyme disulfide bridges, restoring activity (Figure 8).

In Figure 9, two stages in recovering the activity of β-glucosidase in *S. edule* induced by 2-ME are recognized. This activity was inhibited by 0.04 to 12.5 mM Hg2+, confirming the observations made by Anfinesen in 1973 [36]. Enhancement of enzymatic activity with the addition of 2-ME at the above concentrations, can, most likely, be attributed to the appropriate arrangement of the native enzyme. At the second stage, concentrations, ranging from 0.78 to 12.5 mM Hg2+ inhibited different grades of enzyme activity, with 0.1 mM 2-ME recovering just 45% of initial enzymatic activity.

The association between cysteines on enzymes and mercury compounds was extremely strong and had a powerful inhibitory effect, as reported by Waku *et al.* [38]. That fact that Hg2+ was seen to inhibit β-glucosidase suggests that mercury compounds cause dissociation into sub-units or denaturizing protein. The presence of cysteine residues in the active site and the interaction of Hg2+ with SH groups is so strong, that the enzyme-substrate complex cannot be easily separated. For this reason, the inhibition of β-glucosidase in *S. edule* is notable at low concentrations, as the lack of reactivation suggests a sub-unit disassociation (as previously discussed). It is composed of two monomers of 58 kDa, which alter its three-dimensional structure, and therefore, its activity.

The inhibition of β-glucosidase by Hg2+ may be explained as a result of mercury compounds, inducing the dissociation and denaturalization of protein sub-units. The existence of cysteine residues in the active site of β-glucosidase in *S. edule*, and the interaction of Hg2+ with SH groups on this site, is extremely powerful, inhibiting the separation of the enzyme-substrate. 

β-Glucoside in *S. edule* can have cysteines located on the surface of the enzyme, although, not in this case, as iodoacetamide apparently requires a period of 24 to 72 h to take effect. These cysteines are located in a deeper region of the enzyme, similar to the active site of other β-glucosidase cysteines, which are blocked in the presence of iodoacetamide [35], and resulting in a significant loss of more than 50% of initial enzyme activity. Inhibition was enhanced (Figure 11) when the enzyme was pre-incubated with 1 M urea for 30 min at room temperature. Iodoacetamide was then added, using concentrations of 1.56 to 50 mM. There was a 90% decrease in activity with a consequent denaturizing of the protein, resulting in a loss of activity. This may be explained as exposure to intermolecular cysteines, which are blocked by the alkylating agent, prohibiting the formation disulfide bridges, and resulting in a further decrease in activity of *S. edule* β-glucosidase.

In some/certain cases, β-glucosidase is associated with a lectin, as in grasses [39] previously identified as BGAF (aggregating factor β-glucosidase) in a molecular complex. This phenomenon has not been identified in β-glucosidase in *S. edule*, *i.e.*, the presence of lectin obtained from the exudates of the vegetable [40]. Therefore, a more extensive study of β-glucosidase is necessary to understand *S. edule* and the complete sequence of this new β-glucosidase, and to encounter the native structure and its possible role in this vegetable.

### 2.5. Concentration-Dependent stability of β-Glucosidase in S. edule 

**Figure 12:**
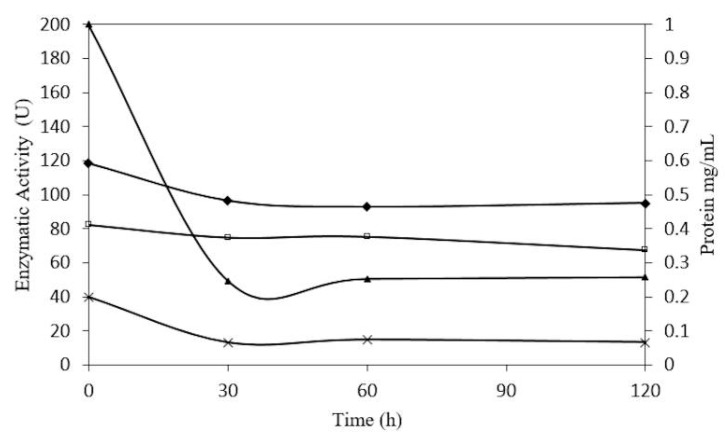
Concentration-dependent stability of β-glucosidase from *Sechium edule* at 45 °C, in pH 5.

In relation to the stability of β-glucosidase in *S. edule*, shown in (Figure 12), an initial concentration of 0.2 mg/mL showed a 33% decrease in protein concentration at pH 5.0, over a period of 30 h, and was stable but active for 5 days. However, 1 mg/mL of β-glucosidase showed a 75% decrease in protein concentration over 30 h and but was stable for 5 days. At 4.0 pH the trend in reduction was similar.

In stability tests, the precipitate was centrifuged and quantified, showing increases with incubation times. These results were similar to the findings of Rosales-Calderon *et al.* [41], who proposed that temperature promotes aggregation and precipitation, explaining the initial decrease in protein. It can be assumed that β-glucosidase in *S. edule* is stable under the conditions indicated above.

### 2.6. Kinetic Parameters of β-Glucosidase in S. edule

**Figure 13:**
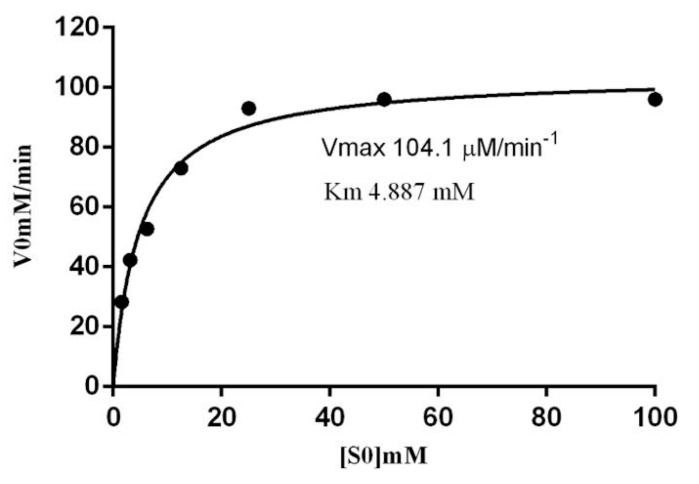
Michaelis-Menten enzyme kinetics. *K*_m_ and V_max_ for β-glucosidase in *S. edule* with *p*-nitrophenyl-β-d-glucopyranoside as substrate.

The kinetic parameters of β-glucoside in S. edule show a *K*_m_ value of 4.88 mM. (Figure 13). The V_max_ value obtained for β-*p*-nitrophenyl-β-d-glucopyranoside under standard assay conditions was 104.1 μM/min^−1^. The *K*_cat_ was 10,000 min^−1^. The catalytic efficiencies of β-glucoside in *S. edule*, given by the *K*_cat_/*K*_m_ ratios (2046 min^−1^/mM) were similar to other β-glucosidases than have been reported previously.

*K*_m_ is a measure of the affinity of the enzyme for its substrate, therefore it depends on the type of substrate, among others variables, such as is reported by other β-glucosidases [42].

### 2.7. Inhibition Assays of β-Glucosidase in S. edule

**Figure 14:**
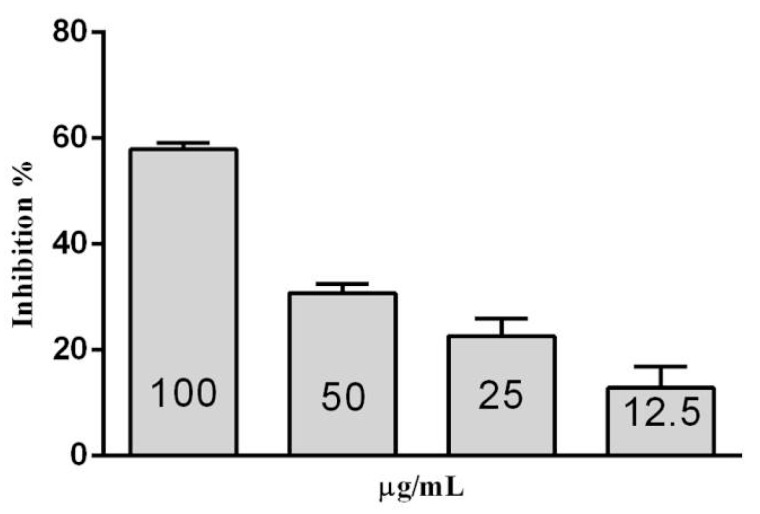
Inhibition of β-glucosidase in *S. edule* from β-glucose at 90 min incubation.

In (Figure 14), β-glucose is shown to inhibit β-glucosidase in *S. edule* at 12.5 to 100 μM. Activity was evaluated every 15 min to 90 min. 15% inhibition is indicated with 12.5 μΜ β-glucose, and 58.8% with 100 μM. 50% inhibition corresponds to 85 μM, with 200 mg/mL Deoxynojirimycin inhibiting 90% of activity.

For this reason, 100 μM β-glucose is perceived as a less effective inhibitor than 100 μM deoxinojirimycin, indicating that high concentrations may be required to inhibit the newly studied β-glucosidase, may be beneficial for its efficiency in commercial use. 

With the aim of solving and preventing the problem of dissociation, and reducing multimeric enzyme inhibition, various strategies, such as, use of chemical cross-linking, protein engineering, or immobilization of enzymes [20,43,44,45,46] were used.

Multimeric enzyme inactivation was not only caused by subunit dissociation, but also had other causes, the information for which is published elsewhere [20,47,48,49].

## 3. Experimental Section

Chayotes (*S. edule*) from the Central Valley of Oaxaca were collected after four months growth, and classified by INIFAP-Oaxaca. They were washed with distilled water and peeled.

### 3.1. Protein Precipitation

Two hundred g of the pulp (epicarp and mesocarp) was mashed and homogenized (2:1 *w*/*v*) using an acetate buffer, 200 mM NaAc, pH 5.0 (extraction buffer). The crude extract was then filtered and centrifuged at 10,000 *g*, (30 min, 4 °C) and the supernatant was adjusted to pH 4.6 by adding (*v*/*v*) glacial acetic acid, and it was incubated for 24h at 4 °C. The resulting precipitated protein was centrifuged and resuspended in the extraction buffer mentioned above. The protein concentration was determined by the Bradford method, using bovine serum albumin as a standard [50], and the enzymatic activity was measured, as mentioned in Section 3.6 [34].

### 3.2. Enzyme Purification

#### 3.2.1. Cationic Interchange Chromatography

Purification was conducted in two stages. First, 50 mg of resuspended protein was poured onto a cationic interchange carboxymethylcellulose column, previously equilibrated with 200 mM NaAc, pH 5.0; at room temperature. Unbounded molecules were washed to baseline absorbance giving an optical density (OD_280)_ of less than ≤0.01. The enzyme was then eluted using a phosphate buffer (PBS, 0.2 M) of pH 6.8, 6.9, 7.0 and 7.1. During purification, 1 mL fractions were collected at a flow rate of 35 mL/h; protein concentration [50] and enzymatic activity [23] was measured in each fraction.

#### 3.2.2. Size-Exclusion Chromatography

Fractions displaying enzyme activity from cationic interchange chromatography were concentrated, and 5 mg of protein was applied to the Sephacryl HR-200 column (Sigma-Aldrich) previously equilibrated with PBS (pH 6.7), and eluted at a flow rate of 16 mL/h, collecting 1.5 mL fractions. Protein concentration and enzymatic activity were quantified for each fraction.

### 3.3. Determination of Molecular Mass

Molecular mass and homogeneity of the purified enzyme were evaluated in 10% polyacrylamide gel electrophoresis (SDS-PAGE) in the presence of 0.1% sodium dodecyl sulfate (SDS), with the Laemmli buffer system [51]. A measurement for native molecular mass was calculated through native-PAGE with 0.05 M phosphate buffer, pH 8.3, according to the Hedricks and Smith method [52]. Gels were stained with 0.1% Coomassie brilliant blue G-250 (Sigma Chemical, St. Louis, MO, USA).

### 3.4. Peptide Mass Fingerprinting

Peptides from purified protein were obtained by Nano-LC-ESI-MS/MS, as described by Alpuche *et al.* [32], after which, protein bands of the purified enzyme from *Sechium edule*, post electrophoresis, were excised from the SDS-PAGE with a sterile scalpel. The gel pieces were washed twice, for 15 min, with 50% (*v*/*v*) acetonitrile in 25 mM ammonium bicarbonate (pH 8.5), in order to remove the Coomassie dye. After dehydrating with 100% (*v*/*v*) acetonitrile for 10 min at room temperature, the gel pieces were vacuum-dried and rehydrated at 37 °C overnight, with sequencing-grade modified trypsin (Promega, Madison, WI, USA) in 25 mM ammonium bicarbonate (pH 8.5). The in-gel triptych digested samples were injected into an integrated Nano-LC-ESI-MS/MS system (quadrupole/time of flight, Ultima API, Micromass, Manchester, UK). The injected samples were first trapped and desalted isocratically on a LC-Packing PepMap C18 μ-pre-column cartridge (Dionex, Sunnyvale, CA, USA). After dissolving the samples with 0.1% formic acid, they were loaded into an analytical C18 capillary column, connected online to the mass spectrometer. Instrumental operation data acquisition and analysis was performed under the full control of Mass-Lynx 4.0 (Micromass). The 1 s survey scans were run through a mass range of *m*/*z* 400 to 2000. The acquired peptide ions were analyzed with the Mascot program (www.matrixscience.com) using both NCBI and EST databases. Parameters for the Mascot search required a peptide mass tolerance of 1 Da, and a MS/MS ion mass tolerance of 1 Da. Variable modifications included methionine oxidation and cysteine carbamido-methylation. Only proteins with significant ion scores (>62) were reported [33].

### 3.5. Bioinformatic Analysis

A search for similarities (Blast) in conserved domains was performed with the highest rated peptides obtained from the MS on the MASCOT computer program (www.matrixscience.com) alignments, with β-glucosidase from *Cucumis sativus*. The sequence analyses were performed using ClustalW2 from the EMBL-EBI program (www.ebi.ac.uk/Tools/msa/clustalo/) [53]. All experiments were made in triplicate, and a one-way analysis of variance was completed for each set.

### 3.6. Enzymatic Activity

Enzymatic activity of the crude extract and all purified fractions was measured using *p*-nitrophenyl-β-d-glucopyranoside (*p*NPG) as a substrate. More specifically, the fractions were diluted (1:100) in sodium citrate (0.2M, pH 5.0). 50 µL was incubated with 50 µL of substrate (5 mM) at 25 °C, for 5 min, in a 96 well microtiter plate. The reaction was stopped with 50 µL of sodium bicarbonate (NaHCO_3,_ 400 mM). The *p*-nitrophenol produced was quantified at 410 nm. (One unit of activity was defined as the amount of enzyme capable of hydrolyzing 1 μM of substrate per hour). Specific activity (SA) was expressed in units of enzyme per mg of protein [23,34]. Protein concentration was determined through the Bradford method at 590 nm.

The Michaelis-Menten model Y = V_max_ × X/(*K*_m_ + X) was used to determine substrate concentrations, which yielded a *K*_m_ (half-maximal velocity) and a *K*_cat_ using nonlinear regression with Prism 6 (GraphPad Software. Inc., San Diego, CA, USA, version).

### 3.7. Effects of pH and Temperature on Enzyme Activity

In order to identify the optimal pH for enzyme activity, purified fractions were dialyzed on buffers, varying the pH from 3 to 9 (acetate buffer 0.2 M, pH 3.5 to 5.5; phosphate buffer 0.2 M, pH 6–8; borate buffer 0.2 M, pH 8.5–9). They were then evaluated with the substrate, as previously described. The optimal temperature for enzyme activity was determined by incubating the enzyme-substrate mixture at intervals of 5 °C, from 0 to 70 °C, for 60 min. Aliquots were withdrawn at regular intervals and cooled in an ice bath, and the remaining activity was calculated through pNPG assays.

Thermostability was evaluated by incubating the enzyme at 200 mM NaAc, pH 5.0 at 30–60 °C at regular periods of 10 to 60 min, all samples were assayed for activity in identical conditions.

### 3.8. Effect of 2-ME, Ions, and Iodoacetamide on Enzyme Activity

One mg/mL purified enzyme was incubated with concentrations of 2-ME varying from 0.03 to 4 M, at 4 °C for 10 min, 90 min, and 24 h. In other, differing concentrations of 0.04 to 12.5 mM of MgCl_2_, ZnSO_4_, HgCl_2_ and AgNO_3_ were incubated with 1 mg/mL purified ezyme at 4 °C for 30 min. These were then incubated with 100 mM of 2-ME. 

The effects of blocking enzymatic activity with alkylating agents was studied using iodoacetamide, in view of the fact that metal ions indicate the necessity of a free sulfhydryl group(s) [34]. Iodoacetamide was evaluated by incubating the purified enzyme (1 mg/mL) with various concentrations, of 0 to 50 mM, with 100 mM Tris-HCl, pH 8.5 for 30 min, 24 h, and 72 h. The experiments were done with and without 1 M urea, for 30 min at 25 °C at room temperature.

### 3.9. Concentration-Dependent Stability of β-Glucosidase in S. edule 

β-Glucosidase concentrations of 1 and 0.2 mg/mL were used to identify stability, which was evaluated at under pH conditions of 4.0 and 5.0, at 45 °C, over a period of 30 h, showing stability for 5 days. The samples were centrifuged at 10,000 *g* for 30 min, to quantify protein concentrations and enzyme activity, as previously described.

### 3.10. Inhibition Assays of β-Glucosidase in S. edule 

Inhibition of β-glucosidase in *S. edule* was performed in a 96-well microplate (Zhang *et al.* [21,22]) using pNPG as a substrate. The assay used 50 μL of β-glucosidase in *S. edule* with 1.0 unit/mL enzyme activity.

In separate experiments, an initial concentration of 100 μM β-glucosidase from *S. edule* and 50 μL β-glucose were combined, followed by a series of dilutions with the buffer. The samples were then incubated for 30 min at 37 °C. The final step involved the addition of 30 μL of 2 mM pNPG in a sodium citrate buffer at pH 5.6, 0.2 M. The reaction was halted with HCL 1M, obtaining results with the ELISA reader at 410 nm, every 15 min to 90 min, (Stat Fax 2100).

The inhibitory activity was determined by calculating the area under the curve for each sample. The logarithmic method was used to calculate the concentration of β-glucose which had 50% inhibition of enzyme activity.

One hundred μL sodium citrate buffer at pH 5.0 was used as a negative control, and 100 μM Deoxynojirimycin was used as a positive control. 50 μL of 100 μM/mL glucose plus 50 μL of citrate buffer was used as a blank.

## 4. Conclusions

This is a new β-glucosidase, covering 10% of the sequence region of glycosyl hydrolase GH I found in *Cucumis sativus* (cucumber). It shows minor differences in its properties, is thermoresistent, and is not inactivated by 4 M 2-ME. It is inhibited by the cations Ag(+) and Hg2+, in a similar way to other β-glucosidases. The properties observed in β-glucosidase in *Sechium edule* make it a good candidate for potential application in bioconversions, although the identification of its natural substrate remains to be determined.
